# ETH and Burs-α are necessary for the normal molting in *Dalbulus maidis* and *Delphacodes kuscheli*

**DOI:** 10.3389/finsc.2026.1811933

**Published:** 2026-06-05

**Authors:** Nicolás Luján Andrada, Mariana Crespo, Agustin Ariel Baricalla, Lucia Inés Dalaisón-Fuentes, Marcos Sterkel, Maria Inés Catalano

**Affiliations:** 1 Centro de BioInvestigaciones (CeBio−CICBA), Universidad Nacional del Noroeste de la Provincia de Buenos Aires (UNNOBA). Pergamino, Buenos Aires, Argentina; 2 Centro de Investigaciones y Transferencias del Noroeste de la Provincia de Buenos Aires (CITNOBA−CONICET). Pergamino, Buenos Aires, Argentina; 3 Instituto Multidisciplinario de Investigaciones Biológicas (IMIBIO-San Luis), Centro Científico Tecnológico San Luis, Área de Biología Molecular, Departamento de Biología, Facultad de Química, Bioquímica y Farmacia, Universidad Nacional de San Luis, San Luis, Argentina; 4 Research and Development Department (R&D), Rizobacter Argentina S.A., Pergamino, Buenos Aires, Argentina; 5 Centro Regional de Estudios Genómicos – CREG -Universidad Nacional de La Plata, La Plata, Argentina

**Keywords:** ecdysis, maize (*Zea mays* L.), neuropeptide, RNAi pest control, Transcriptomic analysis

## Abstract

**Introduction:**

Insects are among the most diverse and widely distributed organisms worldwide. They are studied for both their beneficial ecological roles and their impact as agricultural pests. Molting is a critical process for insect growth and development and is tightly regulated by specific enzymes and hormones. In this study, we identified and characterized two key hormones, Bursicon-α (Burs-α) and Ecdysis Triggering Hormone (ETH), in *Dalbulus maidis* and *Delphacodes kuscheli*, two significant corn pests in Argentina.

**Methods:**

We performed sequence identification and characterization of Burs-α and ETH, followed by RNA interference (RNAi) experiments to evaluate their functional roles. Gene silencing was assessed by measuring mRNA levels, and survival and molting-related phenotypes were recorded after dsRNA treatment.

**Results:**

Silencing *burs-α* and *eth* significantly reduced their corresponding mRNA levels and caused high mortality rates in both species. Treated insects showed severe molting-related defects, supporting the essential roles of Burs-α and ETH in the regulation of ecdysis and post-ecdysis processes.

**Discussion:**

These findings indicate that Burs-α and ETH are essential for insect survival and successful molting in *D. maidis* and *D. kuscheli*. Therefore, these hormones represent promising molecular targets for the development of innovative pest control strategies in corn production.

## Introduction

Insects are one of the most diverse groups of animals in the world. Their remarkable ability to adapt to changing environmental pressures is crucial to their evolutionary success. One key mechanism behind this adaptability is molting. This process allows them to replace their exoskeletons as they grow, enabling them to survive and thrive in a variety of environments (1, 2). Molting occurs in four highly coordinated phases: (1) apolysis, by which the epidermis detaches from the old cuticle; (2) secretion of a new cuticle and degradation of the old one; (3) ecdysis; and (4) tanning of the new cuticle (3–6). Ecdysis, the third step, is a highly stereotyped behavioral sequence involving coordinated peristaltic contractions, air swallowing, and rhythmic motor patterns that enable the detachment and shedding of the old cuticle (7, 8). During this step, insects are particularly vulnerable to environmental stress and predation. Tanning, the fourth and final step, consists of the melanization and sclerotization of the new cuticle, processes that increase its mechanical strength and protective function (4, 5, 9).

The final two steps (ecdysis and tanning) are regulated by neuropeptides that act as hormones (10–12). The ecdysis-triggering hormone (ETH) and Bursicon (Burs) are two important neuropeptides involved in ecdysis (third step during molting) and tanning of the new cuticle (last molting step), respectively (11, 13–17). ETH is produced by the INKA cells, a group of cells located on the epitracheal glands (18). This hormone plays a central role in triggering and coordinating the ecdysis behavioral sequence (19). On the other hand, Burs is produced by peptidergic neurons in the subesophageal and ventral nerve cord ganglia and acts systemically after molting (11). This neurohormone is essential for cuticle tanning, including melanization and sclerotization of the new cuticle, and for wing expansion in adults (11, 15, 20). It is composed of two subunits, Bursicon-α (Burs-α) and Bursicon-β (Burs-β), which form a functional heterodimer (15). Silencing of *bursicon* subunits and *eth* transcripts has been shown to cause severe developmental defects and high mortality in different insect species (10, 14, 21).

The proper formation and sclerotization of the new cuticle are essential for insect survival and post-ecdysial development, since the cuticle constitutes the main interface between the insect and its environment. This structure represents the primary target of many chemical insecticides that act by contact, and alterations in cuticle composition and tanning are frequently associated with reduced insecticide penetration and resistance (22). Consequently, the neuroendocrine pathways that regulate ecdysis and cuticle maturation, particularly those mediated by ETH and Burs-α, emerge as promising molecular targets for the development of novel and more selective pest control strategies.

*Dalbulus maidis* (De Long & Wolcott) (Hemiptera: Cicadellidae) and *Delphacodes kuscheli* (23) (Hemiptera: Delphacidae) are major pests that act as vectors for different pathogens that affect corn crops. *D. maidis* has been identified as a vector of *Spiroplasma kunkelii, maize rayado fino virus (MRFV)* and *maize bushy stunt phytoplasma (MBSP)* that causes the corn stunt disease (24–27), while *Delphacodes kuscheli* has been linked to the transmission of *Mal de Río IV virus (*28*, *29*).* These diseases can cause significant damage to corn production, resulting in lower yields and economic losses for farmers (30, 31).

While neuropeptides have been extensively studied in holometabolous insects particularly in model species such as *Drosophila melanogaster* and *Tribolium castaneum* (10, 12, 21), their role in hemimetabolous insects remains comparatively less explored (3). Investigating neuropeptidergic pathways is therefore essential for a comprehensive understanding of molting regulation across insect lineages. Given the agricultural importance of *D. maidis* and *D. kuscheli* as major corn pests, this research provides new insights into potential pest control strategies based on neuroendocrine disruption. In the present study, we assessed the function of the genes encoding ETH and Burs-α in both Auchenorrhyncha species using RNA interference (RNAi).

## Materials and methods

### Insect rearing

In our laboratory, two laboratory colonies of *D. maidis* and *D. kuscheli* were maintained on corn (*Zea mays L.*) and oat (*Avena sativa* L.) plants, respectively. The colonies were kept in aluminum-framed cages with a fine *voile*-type nylon mesh and placed in a greenhouse at a temperature of 25 °C and 80% relative humidity, with a photoperiod of 16:8 hours (h) (light: darkness). Under these laboratory conditions, both species exhibit similar life cycles, undergoing five nymphal instars, each lasting 4–5 days before molting to the next stage.

### *In silico* identification of *eth* and *burs*-α

Candidate orthologues of *eth* and *burs-α* were identified from transcriptomic datasets assembled in our laboratory for *Dalbulus maidis* (32) and *Delphacodes kuscheli* (Catalano and Andrada, unpublished). Both transcriptomes were generated from whole-body RNA extracted from pooled individuals representing multiple developmental stages, providing broad transcript coverage across the insect life cycle.

The ETH and Burs-α sequences retrieved from UniProt and NCBI from different species were uploaded on the platform *Galaxy Europe* (33) and tBLASTn (34) searches were performed to identify the candidate orthologues of *eth* and *burs-α*. The best hits from BLAST results were chosen under an E-value < 1 x 10^-4^, identity >35%, and bit score >40%. The identified transcripts were translated using the ExPASy translate tool (35) to predict the protein sequence. The resulting sequences were deposited in GenBank under the following accession numbers: *D. maidis eth* (PZ371507), *D. maidis burs-α* (PZ371508), *D. kuscheli eth* (PZ371509), and *D. kuscheli burs-α* (PZ371510).

Multiple sequence alignments were performed using Jalview v2.11.5.1 (36), implementing the MAFFT algorithm (37) with fast Fourier transform–based alignment strategies and the L-INS-i iterative refinement method. These alignments were subsequently used for phylogenetic inference in BEAST X v10.5.0 (38) under the LG amino acid substitution (39) model with gamma-distributed rate heterogeneity among sites (four rate categories). Branch-specific substitution rates were modeled using an uncorrelated lognormal relaxed molecular clock, and tree inference was conducted under a coalescent prior assuming a constant effective population size. Markov chain Monte Carlo analyses were run for 20 million generations, sampling every 2,000 steps, with convergence assessed in Tracer v1.7.2 (40) and all parameters showing effective sample size values above 200.A maximum clade credibility tree was generated in TreeAnnotator v10.5.0 after discarding the initial 10% of sampled trees as burn-in, with node heights summarized as mean posterior estimates. Trees were visualized and edited in iTOL V6 (41).

### RNA extraction and cDNA synthesis

Total RNA was extracted from total insects using TransZol™ reagent (TransGene) according to the manufacturer’s instructions. For primer testing, a pooled RNA sample was prepared from 20 individuals. For subsequent RT-qPCR experiments, RNA was extracted from three insects per biological replicate and treated with DNase I (Thermo Fisher Scientific) to eliminate genomic DNA contamination. RNA integrity was verified by electrophoresis on 1% agarose gels. First-strand cDNA was synthesized using oligo(dT)_18_ primers and the RevertAid Reverse Transcriptase kit (Thermo Scientific), following the manufacturer’s protocol. The resulting cDNA was quantified using a Qubit™ fluorometer (Thermo Fisher Scientific) and diluted to a final concentration of 6 ng µL⁻¹. Specific primers for *eth* and *burs-α* from *D. maidis* and *D. kuscheli* were designed using Primer3plus (42) and *rpl3* as the housekeeping gene (**Table 1**), with two primer pairs per gene: one for dsRNA synthesis and another for qPCR. All primers were tested for dimerization, efficiency, and amplification of a single product. PCR reactions consisted of an initial denaturation step at 95 °C for 2 minutes (min), followed by 45 cycles of 95 °C for 45 seconds (s), 63 °C for 45 s, and 72 °C for 35 s, with a final extension step at 72 °C for 4min (Taq Pegasus, Productos Bio-Lógicos). Amplicon integrity was verified on 1% agarose gels, and the identity of the sequences was confirmed by Sanger sequencing (Macrogen Inc.).

**Table 1:** Primers used for dsRNA synthesis and RT-qPCR analyses.

*Dalbulus maidis*	Forward 5'-3'	Reverse 5'-3'	Base pairs	Oligo type	Efficiency
*burs-α*	AAGTCCTCCCCTGTCTCCTC	AGCCAACAAAGATAGCTGAGT	578	T7	
	TGTGTGTTTCTTCCCTGATGGC	CCCACAACAGGGCTACCAAC	90	RT-qPCR	104%
*eth*	TCTCCTCCCTCCATAGCGTT	TCACCCCAGGAGCACAGAAT	544	T7	
	GTCCTTGAAAAGGTGCACTCC	ACGCTATGGAGGGAGGAGAA	96	RT-qPCR	106%
*rpl3*	GTAAGGACCCAAGAAGCGAGT	TGGCAACTCAATGGAAACAAGA	226	RT-qPCR	106%
*Delphacodes kuscheli*					
*burs-α*	GTCACACCCGTCATTCATGT	GGGACCCTCATCAGCATATC	326	T7	
	TTCAGATTACACAATTCTCG	ATTGAACAAGAGTAGGATTTG	82	RT-qPCR	105%
*eth*	GAGGTCTTCTAGCGACTGCT	ATGGCTCTCTTTTGTGGAACA	394	T7	
	ACATCCGTCTCTACTTCTGGA	ACACGGTCGGATAATTGAACT	116	RT-qPCR	108%
*rpl3*	TGACGGGTTTCAGTGAGA	GGTTCACATTGCAGCGTT	201	RT-qPCR	103%

### RNAi experiments

The neuropeptide precursors analyzed in this study display distinctive sequence features that facilitated transcript identification and RNAi design. *eth* and *burs-α* transcripts were identified based on their conserved peptide motifs and characteristic precursor structure. For dsRNA and qPCR primer design, gene-specific regions were selected and evaluated by BLASTn searches against the corresponding species-specific transcriptomic datasets. No significant similarity with non-target transcripts was detected, supporting the specificity of the selected regions. The results of these analyses are summarized in **Supplementary Table 1** and **Supplementary Figure 1**. dsRNA targeting *eth* and *burs-α* was synthesized using T7 RNA Polymerase (ThermoFisher) according to the manufacturer’s specifications. The same sense and antisense primers containing the T7 promoter sequence at the 5’ end were designed for *in vitro* transcription. dsRNA was purified by DNase digestion (ThermoFisher) and examined on a 1% agarose gel to ensure its integrity. A negative control was included using the *βlactamase* (*β*lac) gene amplified from a pRSET B plasmid (43, 44). The final concentration of all dsRNAs was 0.5 µg/µL.

dsRNA delivery was performed via microinjection into newly molted fifth-instar nymphs (*n*=40 per treatment) as described in (43). Each nymph was injected with 0.25 µL dsRNA at a concentration of 500 ng/µL. After microinjection, nymphs were placed in individual cages attached to maize or oat plants to allow feeding. Insects were monitored daily from injection until completion of molting or death. Particular attention was given to the period corresponding to the expected onset of ecdysis (approximately four to five days after treatment), when phenotypes were examined and photographed.

Phenotypic outcomes were recorded according to the predominant abnormality observed after each RNAi treatment. Because the defects were highly treatment-specific, no artificial severity scale was applied. Individuals injected with ds-*burs-α* were scored as affected when they showed post-ecdysis defects, mainly unexpanded or deformed wings. In a smaller proportion of insects, these abnormalities were accompanied by retention of portions of the old exuviae. Individuals injected with ds-*eth* were scored as affected when they failed to successfully complete ecdysis and remained fully or partially enclosed within the old exuviae.

### RT-qPCR analyses

RT-qPCR experiments were carried out to determine gene expression levels following dsRNA delivery. The fourth day post-injection was selected for RT-qPCR analysis because this time point corresponds to the period immediately preceding the onset of molting-related behaviors in fifth instar nymphs under our laboratory conditions. Individuals of both species typically initiate ecdysis between days 4 and 5 after treatment, making day 4 an appropriate biological time point to evaluate transcript knockdown before phenotypic manifestation. Only live insects were collected for qPCR analyses.

For each biological replicate, three nymphs were collected four days after injection. A total of three biological replicates (*n*=3 nymphs each) per gene (*burs-α, eth, and βlac)* were analyzed. Reactions were performed in technical triplicates (three wells per cDNA sample) in a final volume of 10 μL using Bio-Rad SsoAdvanced Universal SYBR Green Supermix and a Bio-Rad CFX96 thermocycler. Cycling conditions consisted of 30 s at 95 °C, followed by 40 cycles of 15 s at 95 °C and 35 s at 60 °C (annealing and extension).

A melting curve analysis was performed at the end of each run using default instrument settings (65–95 °C, 0.5 C temperature increments). No-template controls were included in all batches. Relative transcript levels were calculated using the ΔΔCt method, as described by (45). Expression values were normalized against the *rpl3* housekeeping gene in both insect species, selected after preliminary evaluation of several candidate reference genes due to its stable amplification performance.

### Statistical analysis

All statistical analyses were performed using GraphPad Prism v8.0.2 (*GraphPad Software, San Diego, CA, USA*). Survival differences among treatments were analyzed using a Chi-square test (p < 0.05). For RT-qPCR data, normality assumptions were not met; therefore, a Kruskal–Wallis test was performed, followed by Dunn’s multiple comparison test for pairwise comparisons.

## Results

### In silico search

**Figure 1:**
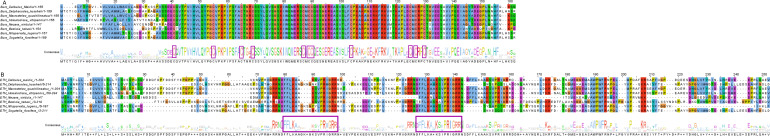
Multiple amino acid sequence alignment of Burs-α and ETH-related peptides in hemipteran insects. **(A)** Alignment of predicted Burs-α amino acid sequences and **(B)** alignment of ETH precursor sequences from hemipteran species, including *Dalbulus maidis* and *Delphacodes kuscheli*. Conserved amino acid residues are highlighted in purple boxes. Consensus sequences are shown below each alignment. Amino acid position numbers are indicated above the sequences.

**Figure 2:**
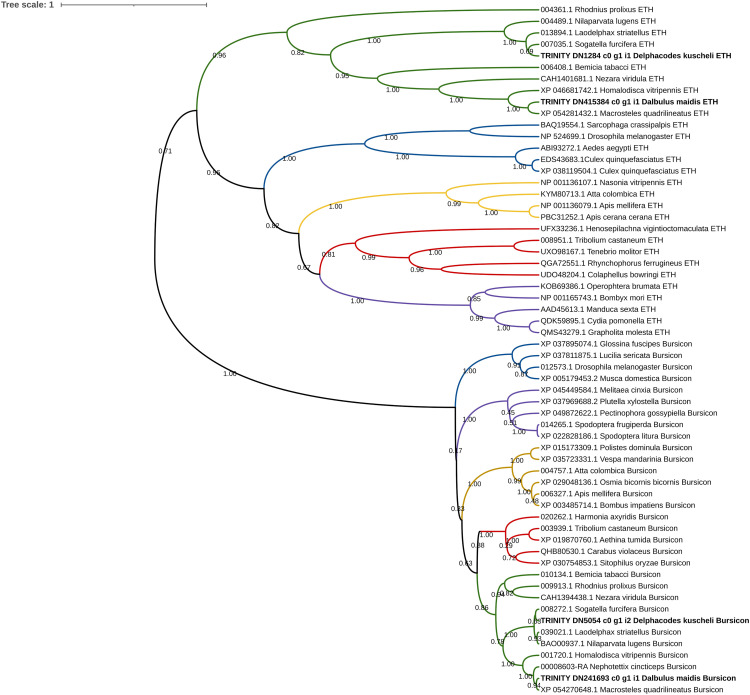
Phylogenetic relationships of ETH and Burs-α sequences in insects. Bayesian phylogenetic tree inferred using BEAST from ETH and Burs-α amino acid sequences of representative insect species. Sequences identified in *Dalbulus maidis* and *Delphacodes kuscheli* are highlighted in bold. Branch colors indicate insect orders: Hemiptera (green), Diptera (light blue), Hymenoptera (orange), Coleoptera (red), and Lepidoptera (purple). Numbers at nodes represent posterior probability values. Illustrations depict representative species from each order.

Orthologous molting-related genes, *eth* and *burs-α*, were identified in *D. maidis* and *D. kuscheli* through *in silico* analysis. The corresponding nucleotide sequences were translated into amino acids and compared with homologous sequences from other insect species in a multiple sequence alignment (**Figure 1**). This comparison revealed a high degree of similarity with orthologs from closely related taxa, further confirming their identity. The conserved *burs-α* sequence contained 11 cysteine residues (**Figure 1A**), while *eth* exhibited the motif FFLKAXXSXPRIGRR (**Figure 1B**). Subsequently, phylogenetic analyses were performed to assess the distribution of these sequences among different insect orders. The resulting tree (**Figure 2**) showed that the analyzed sequences clustered with other cicadellid and delphacid sequences, respectively, consistent with their taxonomic relationships.

### Expression levels after RNAi administration

**Figure 3:**
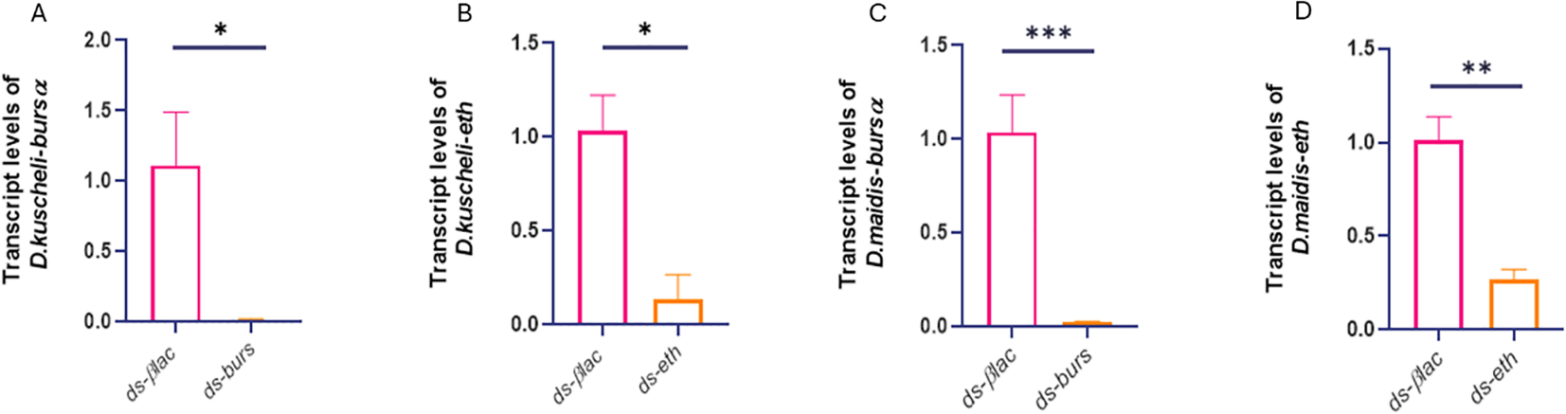
Expression levels of *burs-α* and *eth* in *Delphacodes kuscheli* and *Dalbulus maidis* after RNA interference. Relative transcript levels of *burs-α* in *D. maidis* **(A)** and *D. kuscheli* **(C)**, and *eth* in *D. maidis* **(B)** and *D. kuscheli* **(D)**, measured four days after dsRNA injection. Insects were treated with dsRNA targeting *burs-α* or *eth* (orange bars), or dsRNA targeting βlactamase (ds-βlac; pink bars) as a control. Expression values were normalized relative to the ds-βlac control. Bars represent mean ± SEM of three biological replicates. Statistical significance was assessed using the Kruskal–Wallis test followed by Dunn’s multiple comparisons test. Asterisks indicate significant differences relative to the control treatment [*p* ≤ 0.05(*); p < 0.01 (**); *p* < 0.001(***)].

To evaluate the role of *eth* and *burs-α* in regulating molting behavior, RNAi assays were conducted in both *D. maidis* and *D. kuscheli*. RT-qPCR analysis confirmed strong transcript downregulation following dsRNA treatment. In *D. maidis*, *burs-α* and *eth* expression decreased by approximately 95% and 90%, respectively, whereas in *D. kuscheli*, transcript levels were reduced by about 95% for *burs-α* and 80–85% for *eth* compared with ds-*βlac* control insects (**Figure 3**). Following gene silencing, insects were monitored daily for survival, molting success, and phenotypic abnormalities.

### Phenotypic effects of RNAi

**Figure 4:**
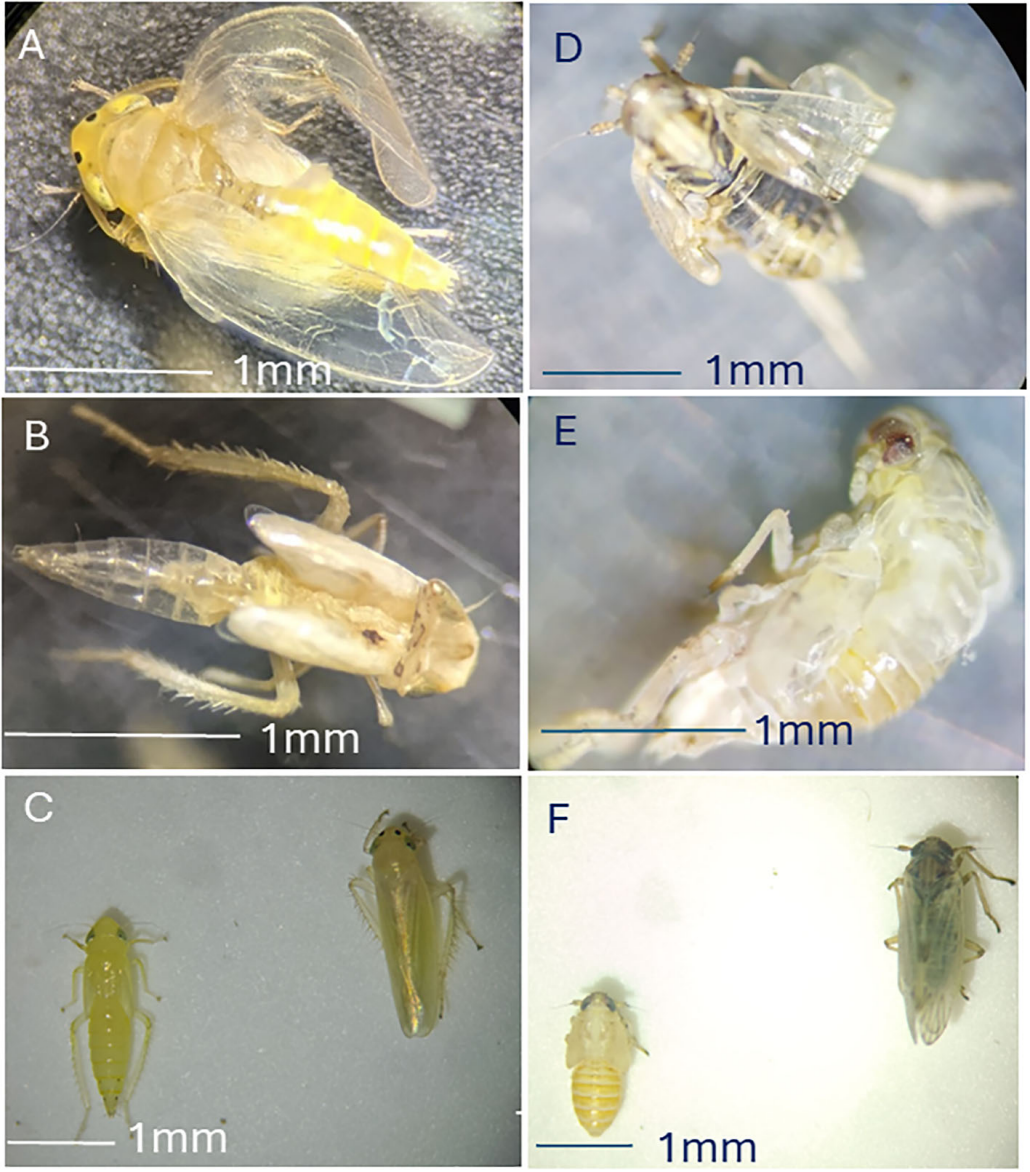
Effects of *eth* and *burs-α* silencing on molting phenotype. Representative images of *Dalbulus maidis* **(A–C)** and *Delphacodes kuscheli* **(D–F)** four days after dsRNA injection. **(A, D)** ds-burs-α-treated insects showing wing deformities associated with unsuccessful ecdysis. **(B, E)** ds-*eth*-treated insects showing incomplete ecdysis. **(C, F)** ds-βlactamase control insects displaying normal molting.

**Figure 5:**
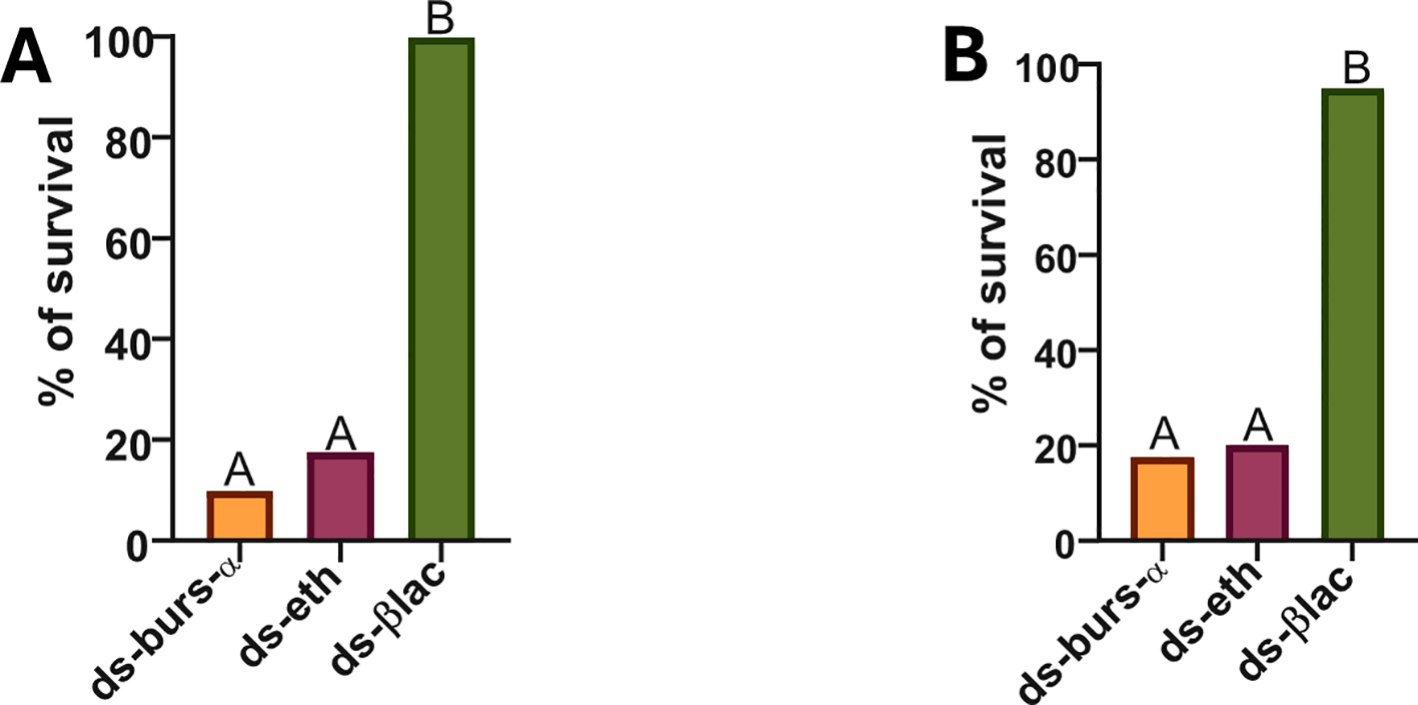
Effect of dsRNA-mediated gene silencing on insect survival. Survival of fifth instar nymphs of *Dalbulus maidis* **(A)** and *Delphacodes kuscheli* **(B)** four days after injection with *ds-burs-α* or *ds-eth*, compared with the ds-βlactamase control (*n* =40). Survival was evaluated at day 4 post-injection, corresponding to the developmental period immediately preceding ecdysis under our laboratory conditions. Different letters indicate statistically significant differences among treatments (Chi-square test, p < 0.05).

*burs-α* silencing caused severe developmental defects in both species (**Figure 4**). Survival decreased markedly within four days after *ds*-*burs-α* injection, dropping to 10% in *D. maidis* and 17.5% in *D. kuscheli* (**Figures 5A, B**). Affected insects displayed severe post-ecdysis defects associated with unsuccessful molting. The predominant phenotype consisted of individuals with unexpanded or deformed wings, observed in 77.5% of *D. maidis* and 75% of *D. kuscheli* insects (**Figures 4A, D**). A smaller proportion additionally retained portions of the old exuviae attached to the body (12.5% and 7.5%, respectively), as shown in **Supplementary Figure 2**. All affected individuals died shortly after the onset of ecdysis, generally within four days after dsRNA injection. In contrast, control insects completed molting normally and showed high survival rates (>95%) five days post-injection (**Figures 4C, F**).

*eth* silencing also produced severe molting defects in both species. ds-*eth*-treated insects failed to undergo successful ecdysis, remaining fully enclosed within the old exuviae without visible emergence. In some individuals, the molting process was initiated but could not be completed, resulting in insects remaining partially enclosed within the exuviae and dying shortly afterward (**Figures 4B, E**). Only 10% of *D. maidis* and 20% of *D. kuscheli* individuals completed molting successfully and remained alive five days after injection (**Figures 5A, B**), indicating that the majority of treated insects were unable to complete ecdysis. These phenotypes were consistently observed among affected insects, and no distinct morphological subcategories were identified. Control insects molted normally, showed high survival rates (>95%), and exhibited no visible abnormalities.

A comparative analysis between both species revealed broadly consistent responses to gene silencing. In both *D. maidis* and *D. kuscheli*, RNAi targeting *eth* and *burs-α* resulted in severe disruption of the molting process, characterized by incomplete ecdysis and high mortality.

However, slight differences were observed in survival rates, with *D. kuscheli* showing marginally higher survival than *D. maidis* following gene silencing (**Figure 5**). Despite these differences, the overall phenotypic outcomes were highly similar between species, indicating a conserved functional role of these neuropeptides in the molting process of both hemipteran species.

Together, these results demonstrate that both genes, *burs-α* and *eth*, are essential for successful molting in both species. Silencing of either gene severely disrupts the ecdysis process, leading to lethal developmental failure. Wing deformities were observed only in insects that partially completed ecdysis, indicating that these abnormalities represent secondary consequences of unsuccessful molting.

## Discussion

Nearly half of the species in the order Hemiptera belong to the suborder Auchenorrhyncha, most of which are phytophagous insects widely distributed worldwide, and many of them act as vectors of plant pathogens (46, 47). Among these, *D. maidis* and *D. kuscheli* are of particular relevance in corn production in the Americas. In addition, recent outbreaks of *D. maidis* in Argentina highlighted the urgent need for effective control strategies. In this context, RNAi has proven to be a valuable tool for managing these pests (43, 44).

Molting is regulated by a complex network of enzymes and hormones. Among these, neuropeptidergic hormones play central roles in the regulation and coordination of the molting process. We identified the ortholog of *burs-α* in the transcriptomes of *D. maidis* and *D. kuscheli*. The neurohormone Burs consists of two neuropeptides, Burs-α and Burs-β, that heterodimerize through disulfide bonds. These bonds are enabled by 11 conserved cysteine residues at specific positions (48–50). We identified the 11 characteristic cysteine residues of Burs-α in both insect species, consistent with the conserved Burs structure reported in other insects.

Additionally, we identified ETH in both species. This hormone is translated as a precursor containing the highly conserved motif FFLKAXXSXPRI, which is cleaved at basic GRR sites to produce the mature ETH peptides (19, 51–54). In most species, there are only two such motifs FFLKAXXSXPRI within a single transcript. We discovered that *D. kuscheli*, like *Nilaparvata lugens* and *Sogatella furcifera* (Fulgoromorpha), has two motifs, whereas *D. maidis*, similarly to *Macrostelles quadrilineatus* and *Homalodisca vitripennis* (Cicadomorpha), presents three motifs. To date, the highest number reported is four repetitions in *Bemisia tabaci* (52). While most holometabolous insects such as *D. melanogaster* and *M. sexta* produce two mature *eth* peptides, hemipteran species examined here displayed two to four copies within the same precursor. Similar lineage-specific expansions of neuropeptide precursors have been reported in comparative analyses of insect neuropeptidomes (55–58), suggesting that internal duplication events may contribute to functional diversification of endocrine signaling in Hemiptera.

We observed a strong reduction in transcript levels of both genes after dsRNA treatment compared with the control in both insect species. This marked downregulation confirms the high efficiency of RNAi under our experimental conditions and demonstrates that both *burs-α* and *eth* are highly susceptible to gene silencing. The strong knockdown obtained in both species highlights the value of RNAi as a functional tool to investigate the molecular regulation of molting and supports future studies evaluating its applicability in pest management approaches (14, 21, 50, 59–61).

The neuropeptide *burs-α* plays a crucial role in wing expansion in adults and cuticle sclerotization after each molt (15, 48, 62). Disruption of this gene leads to defects in wing morphology and insect coloration (21, 63). Accordingly, we observed wing expansion abnormalities similar to those described in the aphid, *Aphis citricidus* (64). The wing malformations consisted of misfolded wings that impaired locomotion and, ultimately, led to death. However, unlike previous studies in moths (50), we did not detect obvious alterations in cuticle coloration. This difference may reflect species-specific variation in the relative contribution of Bursicon signaling to cuticle tanning and post-ecdysis maturation.

The other neuropeptide *eth* is known to activate the motor program for ecdysis (6, 65, 66). When this hormone is absent, insects fail to complete molting and eventually die, as has been demonstrated in grasshoppers, moths, flies, and beetles (10, 14, 20, 67). Likewise, silencing of *eth* in *D. maidis* and *D. kuscheli* produced different phenotypes, ranging from individuals showing no signs of molting to others that initiated the process but could not shed their cuticle completely. These defects impaired locomotion and ultimately led to death, similar to the phenotype observed after *burs-α* silencing. The similar phenotypic outcomes observed in both species, as previously reported in *Rhodnius prolixus*, *Phenacoccus solenopsis* and *Aphis citricidus* suggest that the role of ETH and Bursicon-α in molting is highly conserved among hemipteran insects (64, 68, 69). Although minor differences in survival rates were detected, these variations did not alter the overall response to gene silencing, which was characterized by severe disruption of ecdysis and subsequent mortality. Together, these findings support the idea that key components of the neuropeptidergic regulation of molting are functionally conserved between *D. maidis* and *D. kuscheli*, despite species-specific differences in physiology or life history traits.

In this context, our results demonstrate the importance of Burs-α and ETH for successful molting and normal development of the life cycle in both species. The high mortality observed after gene silencing highlights the relevance of these neuropeptides as essential regulators of ecdysis and reinforces the importance of continued research on the neuroendocrine control of molting in hemipteran insects. However, molting is regulated by a broader neuropeptidergic network, and further studies will be necessary to evaluate the effects of silencing additional hormones involved in this process. Moreover, several neuropeptides are known to participate in biological processes beyond molting, including embryonic development and reproduction. Therefore, evaluating the effects of gene silencing on the offspring may provide a more comprehensive understanding of how the neuropeptidergic cascade coordinates insect development across different life stages.

Taken together, these findings identify *burs-α* and *eth* as promising molecular targets for the development of RNAi-based pest management strategies.

Although RNAi-mediated gene silencing was achieved through microinjection in this study, this approach was employed as a reliable experimental tool to investigate the functional role of *eth* and *burs-α* during molting. Importantly, previous work from our laboratory demonstrated that orally delivered dsRNA can successfully induce RNAi responses in *D. maidis* (43), supporting the potential applicability of alternative delivery strategies beyond microinjection. Nevertheless, the practical implementation of RNAi-based pest management strategies will depend on the development of efficient and scalable delivery methods suitable for field conditions. While microinjection represents a reliable experimental approach for functional characterization, it is not directly applicable in agricultural settings. In this context, alternative delivery systems, including artificial feeding assays, plant-mediated RNAi, and other oral delivery approaches, have shown promising results in several insect species (70, 71). However, the efficiency of these approaches can vary depending on species-specific factors such as dsRNA uptake, degradation, and systemic spread. In hemipteran insects, reduced RNAi efficiency has been associated with dsRNA degradation by extracellular nucleases (dsRNases), which can limit the effectiveness of oral delivery (72, 73).

Previous work in *D. maidis* has shown that silencing dsRNases can enhance the RNAi response following oral delivery, suggesting that co-delivery strategies may improve RNAi efficiency in this species (44). Future studies will be necessary to evaluate the feasibility of these delivery methods in *D. maidis* and *D. kuscheli*, as well as to optimize conditions for effective gene silencing in more applied settings.

Together, these findings support the potential of *eth* and *burs-α* as promising molecular targets for the development of RNAi-based strategies aimed at the selective control of hemipteran pests.

## Data Availability

The data presented in the study are deposited in the GenBank repository, accession numbers PZ371507, PZ371508, PZ371509, and PZ371510.

## Supplementary material

Supplementary Table 1 dsRNA specificity analysis based on BLASTn searches against species-specific transcriptomes. For each target gene (*eth* and *burs-α*) in *Dalbulus maidis* and *Delphacodes kuscheli*, the length of the dsRNA fragments is indicated along with the results of BLASTn searches performed against the corresponding transcriptomic datasets. In all cases, a single significant hit corresponding to the target transcript was detected, and no significant similarity with non-target sequences was found, supporting the specificity of the selected dsRNA regions.

Supplementary Figure 1 Nucleotide sequences and RNAi target regions of eth and burs-α. **(A)** *Dalbulus maidis eth*, **(B)** *Dalbulus maidis burs-α*, **(C)** *Delphacodes kuscheli eth*, and **(D)** *Delphacodes kuscheli burs-α.* The regions used for dsRNA synthesis are highlighted in yellow, and qPCR primer binding sites are indicated in green. The selected regions were confirmed to be specific to the target genes based on BLASTn analysis against species-specific transcriptomes.

Supplementary Figure 2 Less frequent phenotypic outcomes observed after *burs-α* silencing. **(A)** *Dalbulus maidis* and **(B)** *Delphacodes kuscheli* individuals showing combined phenotypes, including wing deformity and retention of the old exuviae. These phenotypes were observed in a smaller proportion of individuals compared to the predominant wing deformity phenotype shown in **Figure 4**.
