# Supplementary material for: ETH and Burs-α are necessary for the normal molting in Dalbulus maidis and Delphacodes kuscheli
Source: Front Insect Sci. 2026 Jun 5;6:1811933. doi: 10.3389/finsc.2026.1811933 (PMC13279319; doi:10.3389/finsc.2026.1811933)
Supplement: Supplementary Table 1 — dsRNA specificity analysis based on BLASTn searches against species-specific transcriptomes. For each target gene (eth and burs-α) in Dalbulus maidis and Delphacodes kuscheli, the length of the dsRNA fragments is indicated along with the results of BLASTn searches performed against the corresponding transcriptomic datasets. In all cases, a single significant hit corresponding to the target transcript was detected, and no significant similarity with non-target sequences was found, supporting the specificity of the selected dsRNA regions. [file DataSheet1.zip › Figure S1.PDF]

dsRNA región

qPCR región

A)

>D.maidis_burs

GCTGATGCGCGGTTGCCTTGCTTTGTTTACAAACAAAGTATTTTGATACTTAAAATGTCAT
AACTAAGTTGTTTGTGTATCTGATAGATGTGTGTTTCTTCCCTGATGGCTCAGTGTTTAGTTT
GATGTGAACTTTTTACCTACGGTGTGATAAAAGTGTTGGTAGCCCTGTTGTGGGCGTGCG
TGTTAGCTGGTGACTCCCCGAAGTCCTCCCCTGTCTCCTCCGAGGACTGTCAGGTGAC
CCCCGTAATCCATGTGCTCCAGTACCCCGGCTGCGTGCCTAAGCCTATCCCCTCCTT
CGCCTGTACTGGCCGCTGCAGCAGCTATCTTCAGGTAAGCGGTTCCAAGATTTGGCA
GATGGAGCGATCGTGCATGTGTTGTCAGGAGAGCGGCGAGCGAGAGGCGAGTGTCT
CACTGTTTTGCCCCAAGGCTAAAGCGGGGGAGAGGAAGTTCAGGAAGGTGATCACGA
AGGCCCCGCTCGAGTGCATGTGTCGTCCGTGCACAGGAGTGGAAGAGAGCGCAGTC
GTGCCCCAGGAGATCGCAGGCTACACAGACGAAGGCCCTCTCAACGCACACTTCCA
AAAGTCCCAGTAAACCTCCCTTCATTCTCTCGGTGACCCCTTCAACTTTACAACCTTGTA
AAATACTCAGCTATCTTTGTTGGCTTCTTAATAAAGTGCTAGTTTTTCAAAAAAAA

qPCR primers
- D. maidis burs qPCR Forward primer

TGTGTGTTTCTTCCCTGATGGC
- D. maidis burs qPCR Reverse primer

CCCACAACAGGGCTACCAAC

T7 primers
- D. maidis burs T7 Forward primer

AAGTCCTCCCCTGTCTCCTC
- D. maidis burs T7 Reverse primer

AGCCAACAAAGATAGCTGAGT

B)

>D.maidis_eth

TTTTTTTTTTTATTGATAAATATATTTTATTTACTTTTATGGTACATTAATTGGCATAAAATTACAA
AATTTTAACTGACAGTTACAATAAATAATCTAGATAACTGCTGGAGATCTGTGAATCATACT
TCGGCTTGCAATCTGGGCTCCTCTACTTCTCTATGTAAGTTGATTTTGTAAAACACTAAAC
CATGGTCACTCTCTTCTTGATCTGGTCCTTGAAAAGGTGCACTCCTCTTGAATGCTTGTG
GCATTAGTTCAGGGTTTCGAAACCAAGGCCATGCTGCCAT**TTCTCCTCCCTCCATAGC**
GTTTGTTAGAGCTCCTCGTTTATCAAGTCTATCCTGTCTACGGCCAATCCGGGGCACAG
ACTTGGCAGCTTTGAGGAAGAAATCATTCCTTCTTCCAATTCTTGGGACGGATTTGGCTG
CCTTCAAGAAGAAATCGTTTCTTCTTTCTACCCTTGGAAAGTTCTTGTTAATCTGAGACGG

CTTTACATCGTCACCATCTCTGCGGCCAATCCTGGGAACGGACTTGGCTGCCTTGAGA
AAGAAGTCATTTCTGCGTCCTACCCTGGGTACATTCTTGACTATGTCCCCAAGGTAAGG
GGGTCCAGGCTCGTAAAAACTATCGTCAAACGGATCAAATTGGTCGTCAAACTGGGATT
GTGGACTGGACTCACTGATGGTCAGAAGACTGCAGACCACGACACATTCCAGCAAAA
GAGTGTAGCAAGCCATGGACTTTGGTGTTTTTCTAAGTTATCTGGTTAGAAGTCTTGGGTC
AACTGATTGCCAAGGGGCAGATCCTGGGTTATATTCTGTGCTCCTGGGGTGAGTGACTC
CTGTATGCGGGCTGACACCTGCACCTGCGGTGGGACATAGGGACATGGTCTCTGCAC
AGCCCAGATTTATCATCAGGAAGTCTTCAGGGAAGTGCTTAAAACAAAACATTCAAATGT
CATTCTATCTCTGCAGAAAAATAAGAAAAATAATGTCAGCAAAATTAGGCTCTTTTCAGCT
ACATTAATTGTGATATCAATACAATCCAAGCATTTGGGAGAATGGTTGTAATAATAAG

qPCR primers

- D. maidis eth qPCR Forward primer

GTCCTTGAAAAGGTGCACTCC

- D. maidis eth qPCR Forward primer

ACGCTATGGAGGGAGGAGAA

T7 primers

- D. maidis eth T7 Forward primer

TCTCCTCCCTCCATAGCGTT

>D. maidis eth T7 Reverse primer

TCACCCCAGGAGCACAGAAT

C)

>D.kuscheli_eth

TGCAGACTTCTTCCCATCACCGAGTAAGTCACTCAGCAAAAAACTTCAACTTATTCAAGT
GCATAAAGTACTATACACTCATCCACCAGTTGCATCGCTTTAGATAGTTCATGAGAACAAT
GTATACAGTTCACAATCGATTCGTAACAGAAGGGGTGTTCCTGATACTATCAGCCACATT
CCTAAGCGTGTTCTCACTGCACAGCGAAGAAAGTATCGACTTGCATTCCAAGTACAAC
CGACCAGGAGGTCTTCTAGCGACTGCTGCAGAGGAGCCATCACTACACACAATGCAA
CGCAGAAACGATTTCTTCCTGAAGGTGAACGGAAAAAACGGAAACAAAATACCAAGAA
TGGGCAGACGCAACATTGTCTATCCAGAGAAGAAAGAAGAGCTCGAGAGTCCGTTTGT
GGTAAGCAGGAGGAACGATTTCTTCCTGAAAGCACACAAATCAATACCGAGAATTGGA
CGCAGGAACAGCAATAGTGAGCCACAATCATCGCTGGCTGATGATAATCAAGCAGCCT
ACAATCAACGACTTTCAAAGAAATTTGACTCTGAGGGCATGGAGAAAGCTGAAGCATCA
GCGTGGCCTTGGTTTAGAGCGCCTGGCTTGTATGTTCCACAAAAGAGAGCCATCTATTT
CACAGAAAACCCAGCTTACTATGGACCAGGTGTAATCATGTGGGATACAGACGAACAT
CCGTCTCTACTTCTGGAAGATAGTGAAGCAAATTTCGGAAGTCTTGACTAAGTTTGGATA
AGCAATCGAATTCTCATGAAAAGTCACTCAAAGTTCAATTATCCGACCGTGTTCTTTGTAT
AATTCAGCCTGGCTCTGAAAATTCAATTTGAAAAATAATAATTATATATTTCTACTTTGAGAA
TAAAACTTATATAAAAAGC

qPCR primers

- D. kuscheli eth qPCR Forward primer

ACATCCGTCTCTACTTCTGGA

- D. kuscheli eth qPCR Reverse primer

ACACGGTCGGATAATTGAACT

T7 primers

- D. kuscheli eth T7 Forward primer

GAGGTCTTCTAGCGACTGCT

- D. kuscheli eth T7 Reverse primer

ATGGCTCTCTTTTGTGGAACA

D)

>D.kuscheli_burs

CTCCTGAATCTACGCCATTATTAAAAGCTTCATTGAAGCACCCACAGCCCTTCTCTCTCT
TGTATCAAATAGGAATATAAAAGTGTCTCTATGTATGTTACTTGAATAAAAGTTTGCTGTTTAA
AAAGTACTTAGGTTAAGATTTCAAAATTTAAAATGACCTGCACGATTCAAATATTTATGCAT
GGAGCAGTCGTTCTTGCCATGATGCTGGCTGAGAGCTTCCAGAATGACGCCAAACCG
GCCGGCGCAGTGTCTTCTGATGAGTGTCAGGTCACACCCGTCATTCATGTGCTTCAATA
CCCCGGCTGTGTTCCAAAACCAATACCCTCATTCGCATGTACCGGAAAATGTAGCAGC
TACTTGCAGGTTTCAGGATCGAAAATATGGCAAATGGAAAGATCATGCATGTGCTGCCA
AGAGAGTGGAGAAAGGGAGGCCAGTGTTTCTCTATTCTGTCCGAAAGCCAAACCAGG
AGAGAAAAAATTTCGCAAGGTGAATACAAAAGCGCCTCTTGACTGCATGTGTCGTCCCT
GCACTGGGGTAGAGGAGACTTCAGTAATACCACAAGAAATAGCTGGATATGCTGATGAG
GGTCCCATGAACAACCATTTCAGATTACACAATTCTCGATAGGAATATTAAAAGAATATCA
TTATGATTATTATTTTTACAAATCCTACTCTTGTTCAATCAGAATAGGATTCTGAAGTACGAG
TATAATCAGCATTCATCGACGAAATCGAATTTTAGTAAAGTCTACAAGAAAAAATTATTCTG
TGTATTGAGTACTTATGAGAAGTTGCTGAACTCAGTTCATTCGATGTAAATAATCATCAATTA
ACTTTCAATCAATTGATTTCAAAAATTATATTTTTGAATTACGTTATGGGAACCGCAATGATT
ATAATACTCATAGTCAGAGACTTCAAAAGTCGGAAAAATTTCAGCTCTAGCTACTTACTAA
ATTTATTTTTCTGAACACAACATAAAATTTACTTTGTTGTAAAACAAGTTTGAAAGTTGCGAT
AGAGTTAAGCTATGTGATTACTTTATAGTACAGTGTGTTAGTTTTTTATTTAATCGTTTTTCAG
ATTGGATGATAGTAGATTAACGAATTTGAAAAATCAGTACAAACTCATAAACTTTTCAATGT
CTTTTTTTAAGAGAAGAAATATGAGCTTCAGATACGC

qPCR primers

- D. kuscheli burs qPCR Forward primer

TTCAGATTACACAATTCTCG

- D. kuscheli burs qPCR Reverse primer

ATTGAACAAGAGTAGGATTTG

T7 primers

- D. kuscheli burs T7 Forward primer

GTCACACCCGTCATTCATGT

- D. kuscheli burs T7 Reverse primer

GGACCCTCATCAGCATATC
