# Supplementary figures and images for: ETH and Burs-α are necessary for the normal molting in Dalbulus maidis and Delphacodes kuscheli
Source: Front Insect Sci. 2026 Jun 5;6:1811933. doi: 10.3389/finsc.2026.1811933 (PMC13279319; doi:10.3389/finsc.2026.1811933)

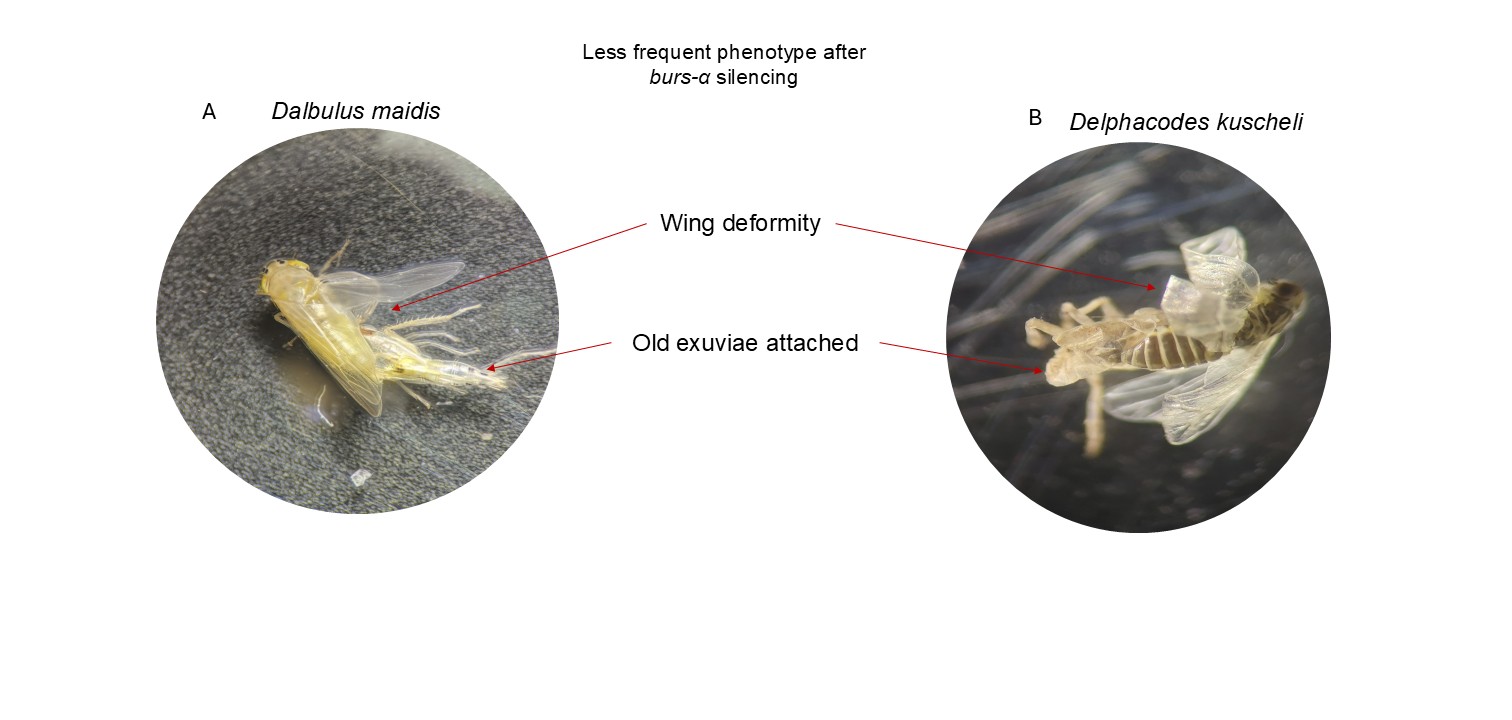

Supplement: Supplementary Table 1 — dsRNA specificity analysis based on BLASTn searches against species-specific transcriptomes. For each target gene (eth and burs-α) in Dalbulus maidis and Delphacodes kuscheli, the length of the dsRNA fragments is indicated along with the results of BLASTn searches performed against the corresponding transcriptomic datasets. In all cases, a single significant hit corresponding to the target transcript was detected, and no significant similarity with non-target sequences was found, supporting the specificity of the selected dsRNA regions. [file DataSheet1.zip › Figure S2.JPEG]

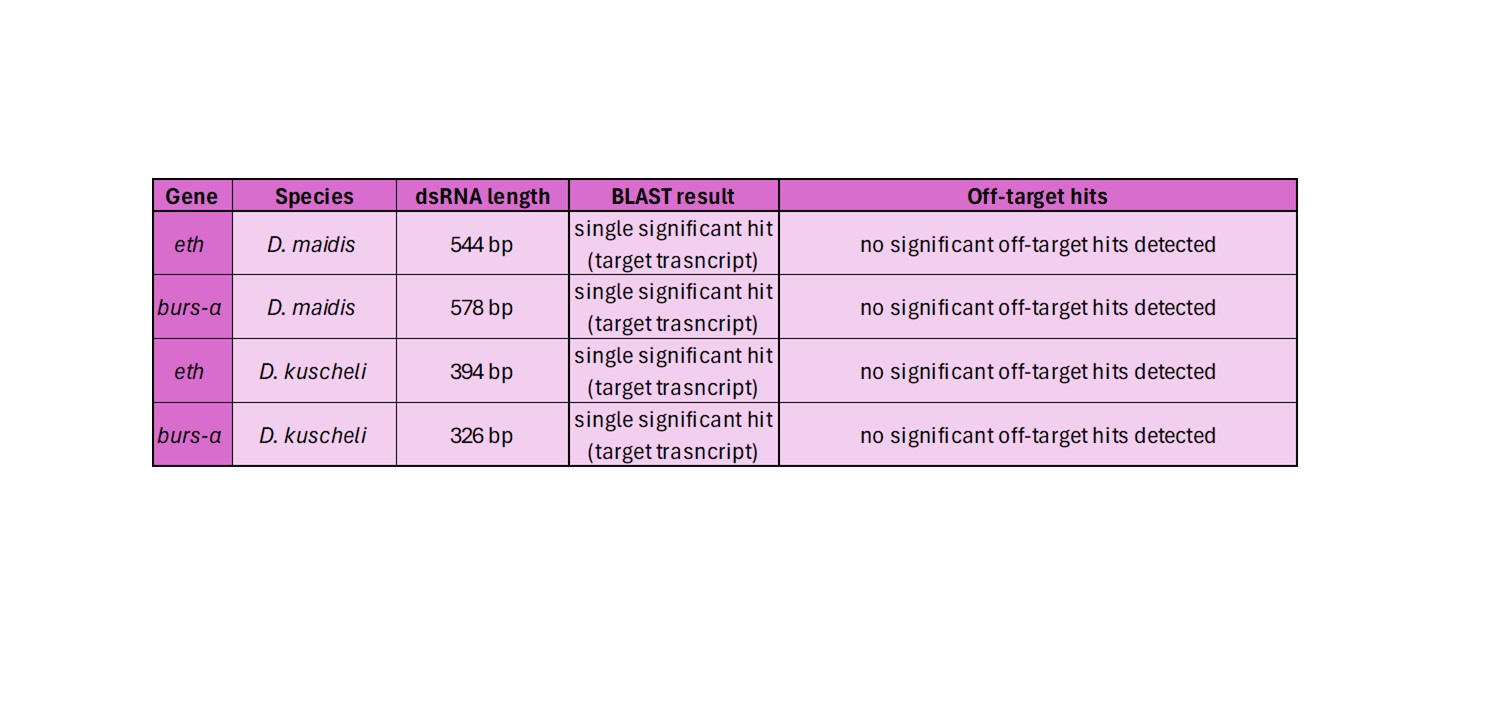

Supplement: Supplementary Table 1 — dsRNA specificity analysis based on BLASTn searches against species-specific transcriptomes. For each target gene (eth and burs-α) in Dalbulus maidis and Delphacodes kuscheli, the length of the dsRNA fragments is indicated along with the results of BLASTn searches performed against the corresponding transcriptomic datasets. In all cases, a single significant hit corresponding to the target transcript was detected, and no significant similarity with non-target sequences was found, supporting the specificity of the selected dsRNA regions. [file DataSheet1.zip › Table S1.JPEG]
